# Modeling the Metabolic State of *Mycobacterium tuberculosis* Upon Infection

**DOI:** 10.3389/fcimb.2018.00264

**Published:** 2018-08-03

**Authors:** Rienk A. Rienksma, Peter J. Schaap, Vitor A. P. Martins dos Santos, Maria Suarez-Diez

**Affiliations:** Laboratory of Systems and Synthetic Biology, Department of Agrotechnology and Food Sciences, Wageningen University & Research, Wageningen, Netherlands

**Keywords:** metabolic model, *Mycobacterium tuberculosis*, systems biology, host-pathogen interaction, condition specific, flux balance analysis

## Abstract

Genome-scale metabolic models of *Mycobacterium tuberculosis* (Mtb), the causative agent of tuberculosis, have been envisioned as a platform for drug discovery. By systematically probing the networks that underpin such models, the reactions that are essential for Mtb are identified. A majority of these reactions are catalyzed by enzymes and thus represent candidate drug targets to fight an Mtb infection. Nevertheless, this is complicated by the limited knowledge on the environment that Mtb encounters during infection. Modeling the behavior of the bacteria during infection requires knowledge of the so-called biomass reaction that represents bacterial biomass composition. This composition varies in different environments or bacterial growth phases. Accurate modeling of the metabolic state requires a precise biomass reaction for the described condition. In recent years, additional insights in the in-host environment occupied by Mtb have been gained as transcript abundance data of interacting host and pathogen have become available. Therefore, we used transcript abundance data and developed a straightforward and systematic method to obtain a condition-specific biomass reaction for Mtb during *in vitro* growth and during infection of its host. The method described herein is virtually free of any pre-set assumptions on uptake rates of nutrients, making it suitable for exploring environments with limited accessibility. The condition-specific biomass reaction represents the “metabolic objective” of Mtb in a given environment (in-host growth and growth on defined medium) at a specific time point, and as such allows modeling the bacterial metabolic state in these environments. Five different biomass reactions were used to predict nutrient uptake rates and gene essentiality. Predictions were subsequently compared to available experimental data. Our results show that nutrient uptake can accurately be predicted. Gene essentiality can also be predicted but accurate predictions remain difficult to obtain. In conclusion, a viable strategy to model Mtb metabolism in hard-to-access environments that is virtually free of pre-set assumptions is provided.

## Introduction

Constraint-based genome-scale metabolic models (GEMs) enable prediction of metabolic states. A metabolic state is defined as a vector of all fluxes or conversion rates (in mmol h^−1^) throughout metabolism per weight unit of biomass (usually 1 gram dry weight, gDw). GEMs comprise linear equations describing conversions among metabolites, uptake or secretion processes, and transport processes over different compartments. These equations are referred to as flux balance constraints and are founded on an underlying metabolic network wherein all metabolites are interconnected by conversion and transport reactions. The flux balance constraints are captured in a stoichiometric matrix (Kauffman et al., 2003). GEMs may comprise additional constraints as well, such as reversibility and capacity constraints. The whole of all possible fluxes that satisfy all constraints of a GEM is referred to as the solution space (Bordbar et al., 2014). Additional constraints present an opportunity to further limit the size of the solution space, which results in a more accurate calculation of the metabolic state. A suitable way to increase the amount of constraints is to measure uptake and/or secretion rates of metabolites/nutrients. Knowledge of a few of these rates can considerably shrink the solution space (Reed, 2012).

Given the stoichiometric matrix, the most straightforward approach for calculating a metabolic state is to simulate conditions wherein the organism is in a steady state physiological condition, meaning that there is no net intracellular accumulation of metabolites. Under this assumption, it is possible to construct a Flux Balance Analysis (FBA) problem. FBA finds the optimal (maximum or minimum) value of a selected function, the so-called objective function, while satisfying all constraints. Solution of the FBA problem leads to a vector of reaction fluxes that represents a calculated metabolic state of the organism. This calculated metabolic state is more likely to represent the actual metabolic state as the solution space is shrunk by additional constraints (Raman and Chandra, 2009; Orth et al., 2010).

The metabolic state is, among others, dependent on the objective function. Metabolic states have been accurately predicted for several bacteria in recent years, using objective functions such as maximizing the flux through the biomass reaction to represent growth rate, maximizing ATP production or minimizing enzyme usage among others (Schuetz et al., 2007).

However, in some conditions measuring uptake and/or secretion rates can be notoriously difficult, if not impossible. Such is the case for intracellular *Mycobacterium tuberculosis* (Mtb), a pathogenic bacterium able to withstand the harsh environment of the phagosome. Mtb is even capable of halting the maturation of the phagosome inside immune cells and providing a niche for the bacterium to thrive (Gengenbacher and Kaufmann, 2012; Zondervan et al., 2018). Genome-scale metabolic models of Mtb, have been envisioned as a platform for drug discovery (Jamshidi and Palsson, 2007; Rienksma et al., 2014).

In addition to uptake rates, other measurements can serve to estimate or approach (a part of) the metabolic state of a cell, such as transcript profiles (Hoppe, 2012). For Mtb, a major difficulty with these measurements is the large size difference between the eukaryotic host cell and the prokaryotic pathogen, which results in metabolites and transcripts from the host vastly outnumbering those of the pathogen (Fels et al., 2017). With regard to transcript abundance experimental methods have been developed to increase the ratio of pathogen mRNA to host mRNA (Mangan et al., 2002). This enrichment in pathogen transcripts renders differences between intracellular and extracellular pathogen transcript abundance apparent. We recently published a dataset of *Mycobacterium bovis* BCG and THP-1 cells using a dual RNA-sequencing strategy (Rienksma et al., 2015). However, such an enrichment method is not available for metabolites, which are more closely related to fluxes as compared to transcripts. Moreover, metabolites, unlike transcripts, cannot be assigned to host or pathogen unless they only occur in one of said host or pathogen (Zimmermann et al., 2017).

Transcript abundance data can be used to constrain models in environments where knowledge regarding nutrient availability and objective(s) is limited. Methods such as iMAT (Shlomi et al., 2008), MADE (Jensen and Papin, 2011), GIMME (Becker and Palsson, 2008), E-flux (Colijn et al., 2009), TRFBA (Motamedian et al., 2017), and others (Lewis et al., 2012) limit the solution space by using expression values as a proxy for flux. These methods allow for the explanation of phenomena that cannot be derived solely from the models, such as the prediction of the Crabtree effect in yeast (Rossell et al., 2013). These model and data integration methods limit the solution space within the ranges of expression data, thereby effectively generating condition specific models. Shrinking the solution space by limiting fluxes based on gene expression seems an obvious choice, but it is not at all obvious how this should be done. Methods for model and data integration have been thoroughly evaluated (Machado and Herrgard, 2014). The evaluation showed that no method outperforms the others for all tested models and datasets. Finally, this condition-specific model building can hamper exploration of metabolic states that arise from perturbations of the environment, from which the gene expression data was originally derived. These adapted models would only allow changes to the metabolic state that fit within the boundaries of what was originally measured. Such a rigid model appears a poor choice for predictive modeling.

A modeling approach focused on an accurate description of the objective of Mtb during infection appears to be a better strategy to make new predictions because it does not limit the solution space or metabolic flexibility beforehand. Previous approaches have relied on adapting the biomass reaction to represent the composition on mycobacterial cells during infection. Bordbar and colleagues adjusted the biomass reaction based on differential gene expression (Bordbar et al., 2010). This approach is biased by the biomass reaction that is present in the model prior to the tailoring process and the potential synthesis of other metabolites specifically during infection, is overlooked. Shi et al. (2010) proposed a biomass reaction comprising trehalose dimycolate, triacylglycerol (TAG), and polyglutamate/glutamine to reflect a minimal cell wall composition. The logical assumption applied was that during a “non-growth state”, Mtb utilizes metabolites produced in pathways of which gene expression is elevated and does not, or to a lesser extent, utilize metabolites produced in pathways of which gene expression is suppressed. Shi and colleagues used qPCR to monitor gene expression (Shi et al., 2010). This requires a pre-selection of target genes based on experience and experimental output and does not accommodate unbiased exploration of the transcriptional landscape.

Here, we integrate a constraint-based model of Mtb metabolism and RNA sequencing data to provide condition-specific biomass reactions during host infection and during growth on Middlebrook 7H9 medium. The genome-scale nature of this approach ensures all known pathways and biomass precursors are taken into account, whereas the nature of the used data (RNAseq) ensures unbiased assumptions on the types and quantities of metabolic precursors. During on-going infection mycobacterial cells might enter a non-growth state on which maximal growth rate is not the metabolic objective. However, still minimal macromolecular components need to be synthesized and energetic requirements need to be fulfilled to ensure survival. The condition-specific biomass reaction representing infection combines both aspects as it reflects the composition of mycobacterial cells during infection, and it also represents the metabolic requirements for its survival and interaction with the host which are incorporated in the RNAseq data as well. To simulate the metabolic state of the bacteria upon infection, flux through the condition-specific biomass reaction is maximized, while the total usage of enzymes is minimized. As Mtb faces several types of stress and adverse conditions imposed by the host's immune system during infection of the host (Fontán et al., 2008), it is assumed that Mtb does not squander its resources, and makes optimal use of available enzymes. From a modeling perspective, this can be seen as a bi-objective optimization problem wherein two competing objectives, i.e., maximization of biomass production on the one hand, and minimization of enzyme usage on the other hand, are simultaneously considered.

The goal of multi-objective optimizations is to find Pareto optimal solutions (also called non-dominated solutions) (Papalambros and Wilde, 2000). A solution is Pareto optimal if no other solution exists that better satisfies all objectives. In other words, a solution is Pareto optimal if an improvement in one objective requires a degradation of another. Multiple methods have been developed to obtain Pareto optimal solutions in multi-objective optimization problems such as the normal constraint method (Messac et al., 2003) that has been used to explore tradeoffs between hepatic metabolic functions (Nagrath et al., 2007). Here, we tackle the problem by using a weighted sum method in which weight factors are attributed to each objective: *f*_*b*_ and *f*_*e, i*_, for biomass and enzyme usage for each of the *i* = {1, …, m} reactions, respectively. This approach ensures that the obtained solutions are Pareto optimal (Suárez et al., 2008). Parsimonious enzyme usage FBA (pFBA) has been proposed to explore the tradeoffs between maximizing growth and minimizing enzyme utilization (Bordbar et al., 2010). In pFBA there is an initial maximization of the biomass reaction followed by a minimization of enzyme usage. In our approach, this Pareto optimal solution would correspond to the extreme case on which the weight of the biomass objective is much higher than that of the enzyme minimization objectives (*f*_*b*_ >> *f*_*e, i*_,). By changing these weight factors, a ratio between these two factors, *f*_*r*_, is established that enables the prediction of metabolic genes essential to Mtb within the macrophage as well as metabolites that are sequestered by Mtb from the phagosome. Comparison of these predictions with experimentally obtained data (Beste et al., 2013; Mendum et al., 2015) reveals that by using a condition-specific objective function inferred from transcript abundance data the metabolic state of Mtb upon infection can be accurately predicted.

## Methods

### Mtb metabolic model

We used our genome-scale metabolic model of *M. tuberculosis* called sMtb, *in silico M. tuberculosis* (Rienksma et al., 2014). We made some minor corrections to this model regarding among others the respiration chain, and added six reactions to improve the functioning of the model. This improved sMtb model can be found in Supplementary Data Sheet 1 (as systems biology markup language file, SBML) and in Supplementary Data Sheet 2 (as excel file) together with a small summary of the aforementioned changes in Supplementary Data Sheet 3. A list of metabolites that could be present in the phagosome was collected from literature (Beste et al., 2013), similarly a list of metabolites in Middlebrook 7H9 medium was collected (Supplementary Table 1).

### Constraining sMtb with gene expression data

Raw sequence read data supporting the results of this article are available in the EMBL-EBI European Nucleotide Archive under the Accession No. PRJEB6552, http://www.ebi.ac.uk/ena/data/view/PRJEB6552 for both *M. bovis* BCG grown on Middlebrook 7H9 medium and *M. bovis* BCG cells infecting THP-1 cells. RNA sequencing reads were aligned to the *M. bovis* BCG genome as described before (Rienksma et al., 2015). For each gene present in sMtb, the number of reads aligning to it was summed. A cutoff value of 100 counts per million (cpm) was used to identify lowly expressed genes, that were assigned a count value of zero. The resulting gene count values were subsequently transferred to their corresponding reactions, summing the counts for reactions catalyzed by isozymes. For reactions catalyzed by a protein complex, the smallest number of counts of every gene that encodes a part of such a complex was assigned to the reaction. For reactions that can be catalyzed by several different protein complexes, the smallest number of counts assigned to one of the genes encoding a part of each complex was identified and subsequently the total of all these smallest numbers of counts was assigned to the reaction. Reactions that received no counts using this method were not allowed to carry any flux. Afterwards, the total number of counts assigned to each reaction was normalized by dividing this total number of counts by the largest number of counts assigned to any reaction in sMtb, resulting in a value ranging between 0 and 1 for each enzyme-catalyzed reaction. This procedure is called the E-flux algorithm and is explained in greater detail by Colijn et al. (2009).

### Obtaining condition-specific biomass reactions

**Figure 1:**
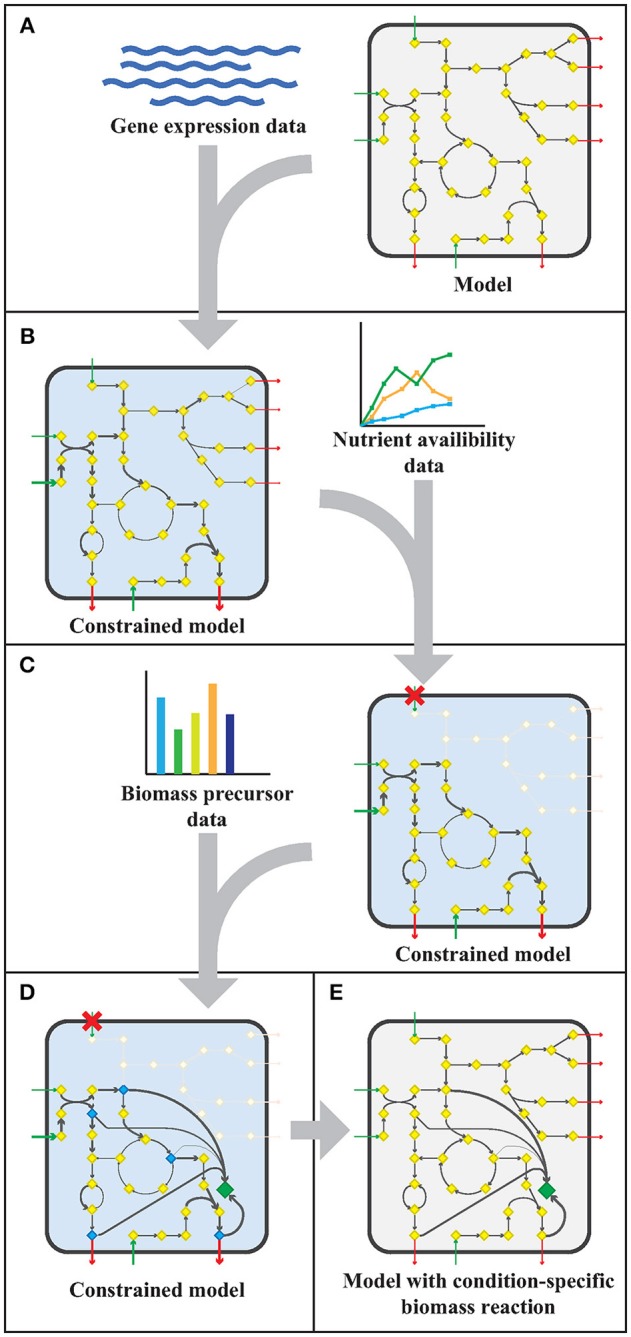
Workflow to create a model with a condition-specific biomass reaction. **(A)** An exemplary constraint-based genome-scale metabolic model (GEM) comprising a metabolic network with metabolites (yellow diamonds) and reactions (arrows), including uptake reactions (green arrows) and secretion reactions (red arrows), is depicted in a microorganism (rounded square). Gene expression data (blue wave-shaped lines) is used to constrain maximal flux values according to the E-flux algorithm (Colijn et al., 2009), to obtain a condition-specific GEM having a shrunken solution space. **(B)** The condition-specific (blue background) GEM is subsequently combined with nutrient availability data (graph) and uptake of unavailable nutrients is constrained to zero. **(C)** Biomass precursor data (bar plot) is used to pinpoint biomass precursors in the condition-specific GEM with blocked transport reactions (red crosses), and the flux through the flux limiting reaction for each precursor is selected by maximizing flux toward each biomass precursor (blue diamonds) individually. **(D)** The sum of all precursor fluxes is normalized to one gram biomass dry weight (1 gDw) and a condition-specific biomass reaction (green diamond) is obtained. **(E)** All constraints placed on the GEM in the previous steps **(A–D)**, are removed and a GEM with a condition-specific biomass reaction is obtained.

The workflow applied to model sMtb is generally depicted in Figure 1. This workflow was applied twice: for gene expression data of *M. bovis* BCG grown on Middlebrook 7H9 medium (medium condition) and for gene expression data of *M. bovis* BCG cells infecting THP-1 cells (infection condition). Firstly, upper bounds on unidirectional (forward) reactions, and upper and lower bounds on bidirectional reactions were replaced by the normalized counts assigned to that reaction (Figure 1A). The resulting sMtb model constrained by gene expression data was further constrained by setting uptake rates of unavailable metabolites to zero in the given condition and allowing unconstrained uptake of available nutrients, based on nutrient availability data (Figure 1B). The nutrient availability in the phagosome and in Middlebrook 7H9 medium is given in Supplementary Table 1. Afterwards, a general list of biomass precursors was obtained from sMtb (Supplementary Data Sheets 1, 2). For each biomass precursor, a sink reaction was added and the flux of each of these sink reactions was individually maximized, effectively maximizing the flux toward the respective precursor (Figure 1C). Subsequently, the obtained maximum value for each biomass precursor was normalized such that the total molecular weight of these precursors equaled one gram, resulting in a condition-specific biomass reaction of Mtb during infection, growing in phagosomal conditions, CSI, and a condition-specific objective function of Mtb growing in Middlebrook 7H9 medium, CSM (Figure 1D). These condition-specific objective functions were subsequently added to sMtb and constraints derived from the gene expression were removed (Figure 1E).

### Calculating nutrient uptake for various objective functions

We compared five different objective functions for their ability to correctly predict nutrient uptake rates by Mtb in the phagosome. The following biomass reactions were used: CSI, CSM, the regular biomass reaction from model sMtb representing growth (REB) (Rienksma et al., 2014), the biomass reaction from model sMtb representing *in vivo* growth (IVB) (Rienksma et al., 2014) and a reaction representing Mtb in a non-replicative state (NRC) (Shi et al., 2010).

**Figure 2:**
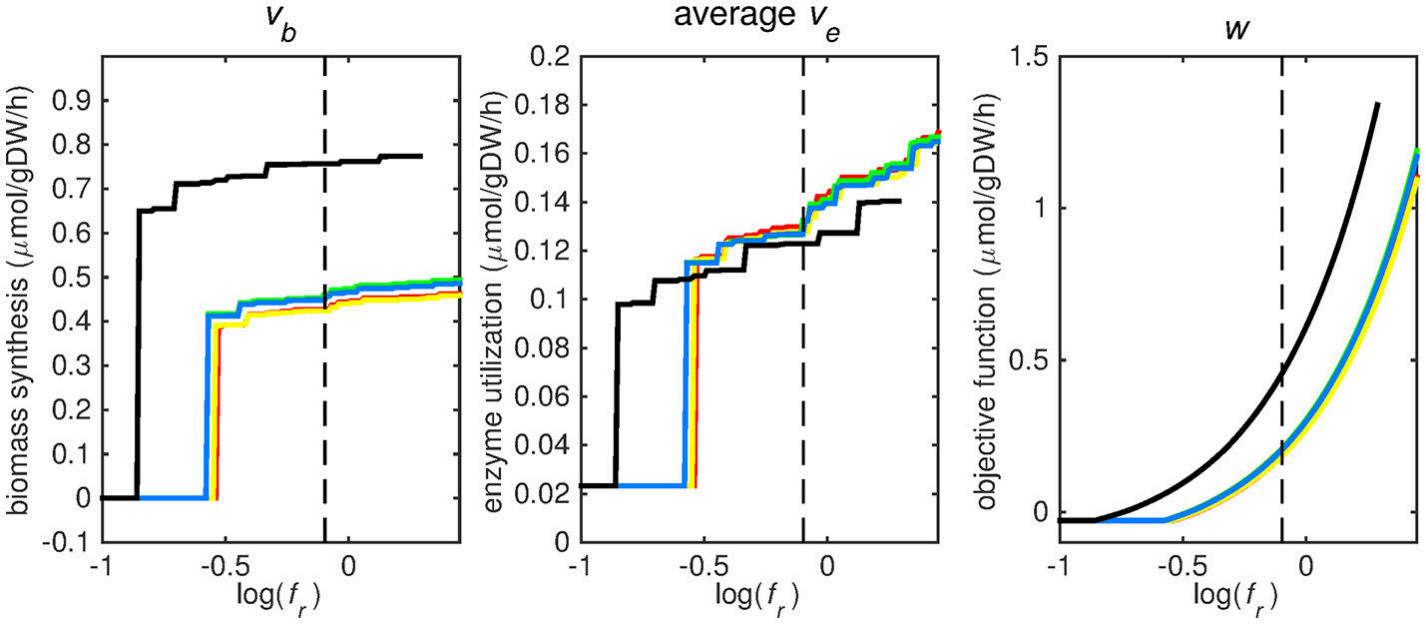
Tradeoff between biomass production (growth rate) and enzyme utilization in the metabolic model. Predicted values of the flux through the biomass synthesis reaction **(left)**, average flux through all enzymatically catalyzed reactions **(middle)** and the objective function value **(right)**, i.e., combination of total enzymatic reaction minimization and biomass reaction maximization for various *f*_*r*_-values. Five different biomass reactions are shown: CSI (green), CSM (blue), IVB (yellow), REB (red), and NRC (black). The dashed line indicates *f*_*r*_ = 0.8.

**Figure 3:**
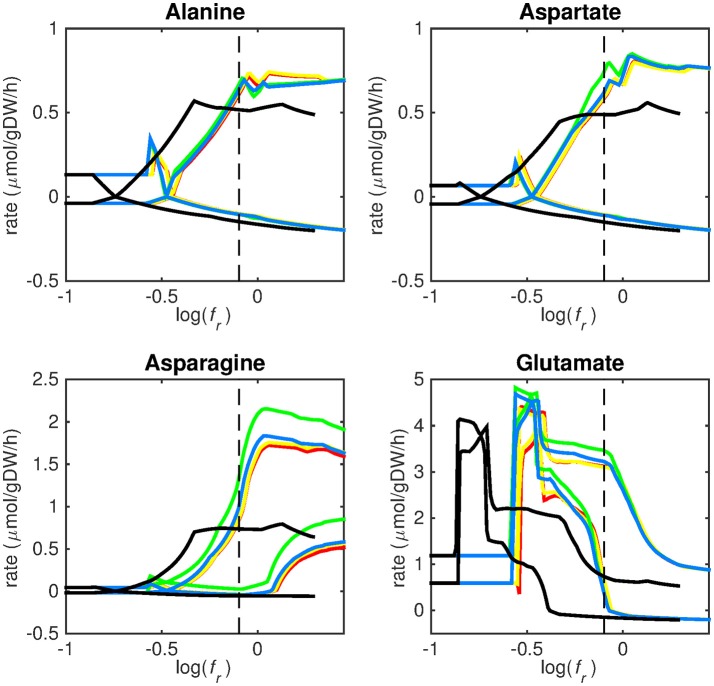
Predicted amino acid uptake rates. Maximum and minimum predicted uptake rates for alanine, aspartate, asparagine, and glutamate using five different biomass reactions: CSI (green), CSM (blue), IVB (yellow), REB (red), and NRC (black) for varying values of the biomass weight factor *f*_*r*_. The dashed line indicates *f*_*r*_ = 0.8. Two lines of the same color indicate upper and lower limits of the prediction. Note that negative values of uptake rates denote excretion of that metabolite.

**Figure 4:**
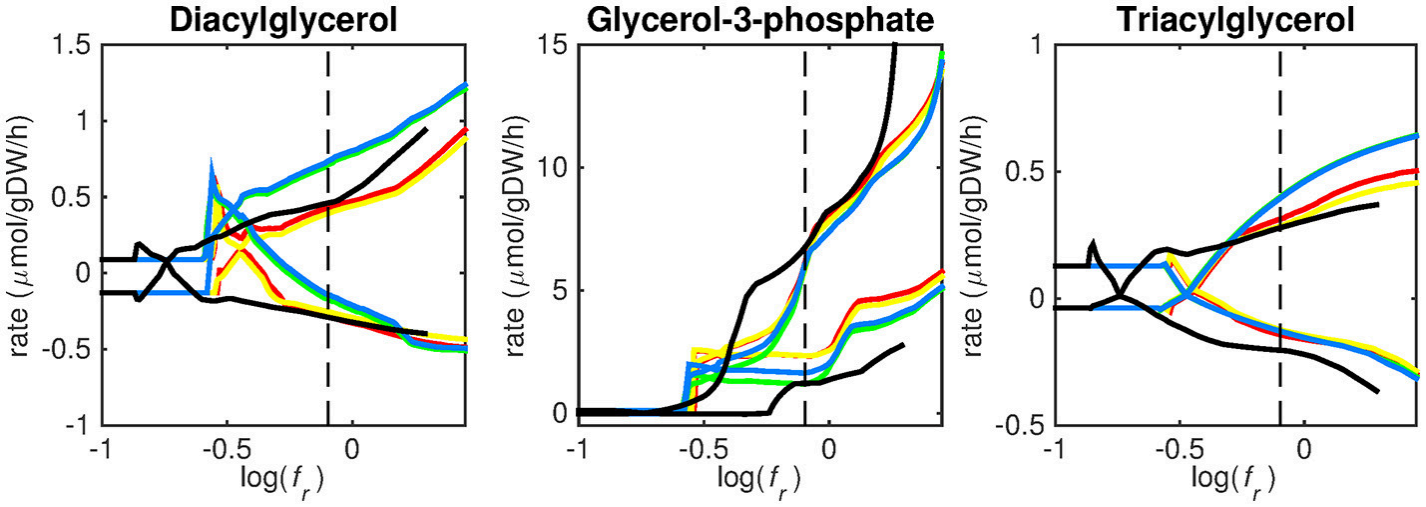
Predicted lipid uptake rates. Maximum and minimum predicted uptake rates for diacylglycerol, glycerol-3-phosphate, and triacylglycerol using five different biomass reactions: CSI (green), CSM (blue), IVB (yellow), REB (red), and NRC (black) for varying values of the biomass weight factor *f*_*r*_. The dashed line indicates *f*_*r*_ = 0.8. Two lines of the same color indicate upper and lower limits of the prediction. Note that negative values of uptake rates denote excretion of that metabolite.

**Figure 5:**
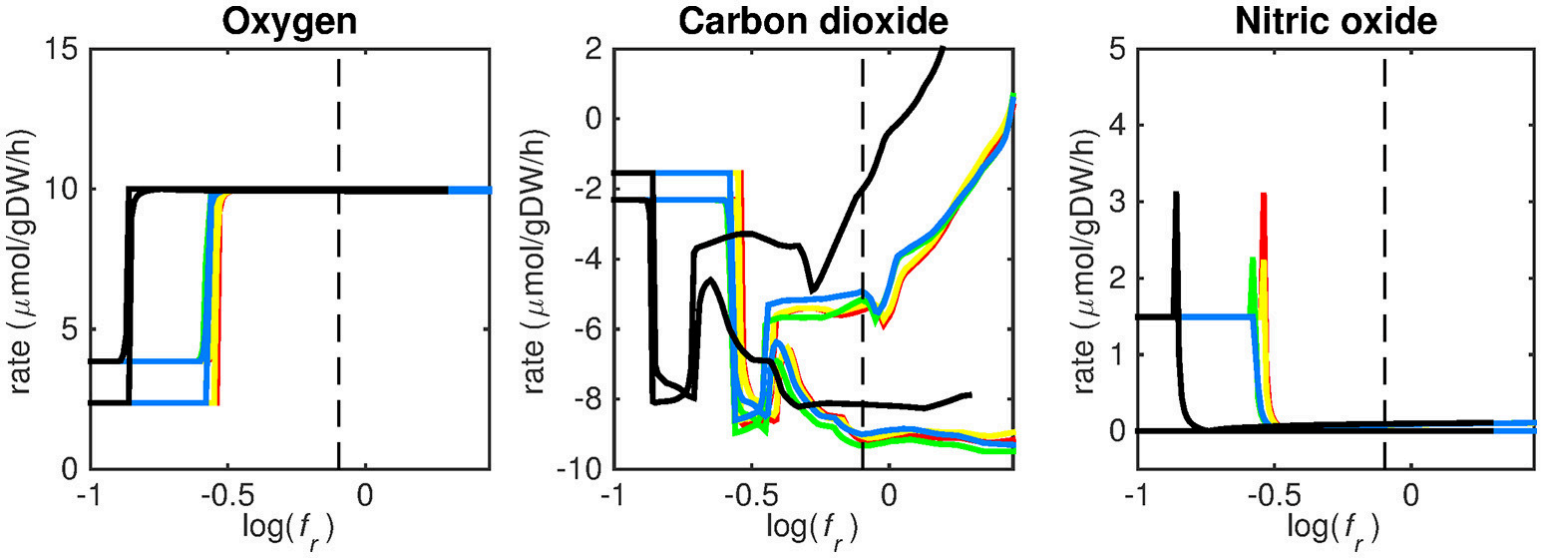
Predicted oxygen, carbon dioxide, and nitric oxide uptake rates. Maximum and minimum predicted uptake rates for oxygen, carbon dioxide, and nitric oxide using five different biomass reactions: CSI (green), CSM (blue), IVB (yellow), REB (red), and NRC (black) for varying values of the biomass weight factor *f*_*r*_. The dashed line indicates *f*_*r*_ = 0.8. Two lines of the same color indicate upper and lower limits of the prediction. Note that negative values of uptake rates denote excretion of that metabolite.

The bounds on uptake rates of all nutrients representing phagosomal conditions (Supplementary Table 1) were unconstrained, with the sole exception of constraining the oxygen uptake rate to 0.01 mmol gDw^−1^ h^−1^. Subsequently, each of the five objective functions was maximized while the sum of all other enzymatically catalyzed reactions was minimized (Equations 1.1 - 1.3). The weight factor for the biomass reaction, *f*_*b*_, was varied while keeping the weight factor for enzymatically catalyzed reactions, *f*_*e, i*_, constant at 0.001 for all reactions *i*, hence effectively varying *f*_*r*_, the ratio between *f*_*b*_ and the sum of all *f*_*e, i*_-values (Equation 2). Subsequently, a flux variability analysis was performed, wherein the maximum objective function value, *w* (Figure 2, right panel), was set as a constraint and the nutrient uptake rates were minimized and maximized individually, resulting in maximal and minimal uptake rate boundaries for various nutrients for each objective function (Figures 3–5) (Gudmundsson and Thiele, 2010; Orth et al., 2010).

### Comparison of gene essentialities for various objective functions

At log(*f*_*r*_) > −0.5 flux is channeled through all (condition-specific) biomass reactions (Figure 2, right panel). At log(*f*_*r*_) < 0.3 minimizing flux through the sum of all enzymatically catalyzed reactions is still relevant and the restrictions of the oxygen uptake threshold are not yet overcome. Beyond this point, the (condition-specific) biomass reaction weight factor, *f*_*b*_, is so large as compared to the sum of all *f*_*e, i*_-values, that an optimal objective function value, *w*, is obtained by solely minimizing enzyme usage, and ignoring maximizing flux through the (condition-specific) biomass reaction. An *f*_*r*_-value of 0.8 was chosen from Figures 2–5 as asparagine, alanine and glutamate in addition to glycerol-3-phosphate and CO2 are taken up from the host, which is likely to occur during infection (Beste et al., 2013) and this value log(0.8) ≈ − 0.1 is centered between the boundaries of log(*f*_*r*_) = 0.5 and log(*f*_*r*_) = 0.3. The growth rates (i.e., the *in silico* calculated flux of CSI, CSM, IVB, REB, and NRC) were maximized indirectly by maximizing the aforementioned bi-objective optimization problem. Each biomass reaction will always obtain its maximal value using this approach. Thereafter, using the COBRA toolbox (Schellenberger et al., 2011), genes and their corresponding reactions where deleted one by one and the resulting specific growth rates were computed by maximizing the aforementioned bi-objective optimization problem. These growth rates were divided by the wild-type growth rate, resulting in a number between 0 and 1 for each knocked-out gene, representing the relative specific growth rate. We applied a 95% reduction in the relative growth rate as a threshold to indicate essential genes as described before (Rienksma et al., 2014).

Mendum and colleagues infected human dendritic cells with an Mtb transposon library to identify genes that are required for *in vivo* survival after 3 days and after 7 days (Mendum et al., 2015). These experimentally identified essential genes were compared to the predicted essential genes using the aforementioned five different objective functions. Subsequently, the accuracy, sensitivity and specificity of the predictions, were calculated for all five objective functions and for both experimental time points.

## Results

We created two condition-specific biomass reactions (CSI and CSM) based on transcript abundance data in two conditions. The term “biomass reaction” is perhaps not the most suitable term as these reactions not only cover synthesis of metabolites used for biomass production, but also synthesis of excreted enzymes and small molecules, repair of damaged lipid membranes and other metabolites involved in host-pathogen interaction. Even though these processes themselves are largely unknown, transcript abundance data indirectly reflects these processes and combined with a GEM can give a picture of required metabolic precursors for these processes.

### Condition-specific biomass reactions

The creation of a condition-specific biomass reaction requires a GEM, a list of available nutrients in the given condition, a list of metabolic precursors for synthesis of macromolecules, and transcript abundance data. We used model sMtb, a comprehensive model of Mtb metabolism (Rienksma et al., 2014), with minor corrections and additions (Supplementary Data Sheets 1–3). Transcript abundance data was obtained from a dual RNA-sequencing experiment wherein transcript abundances of *M. bovis* BCG, a close relative of Mtb having a highly similar genome (Garnier et al., 2003), were measured under two conditions (Rienksma et al., 2015). In the first condition *M. bovis* BCG infects THP-1 cells, and in the second condition *M. bovis* BCG grows on Middlebrook 7H9 medium. The sMtb model was used as a platform to integrate the expression data and to calculate two condition-specific biomass reactions of Mtb, CSI (condition-specific infection) and CSM (condition-specific medium), for both aforementioned conditions, respectively. A list of all metabolites known or expected to be present in the phagosome was assembled (Supplementary Table 1). Availability of these metabolites was simulated by enabling their free uptake in the model. In addition, a list of all known biomass precursors was generated based on the sMtb model (Supplementary Table 2).

The flux toward each biomass precursor was maximized one by one, while limiting the maximum flux through enzymatically-catalyzed reactions based on the transcript abundance for the present condition (Figure 1). The ratio of biomass precursors obtained for both conditions represents the two condition-specific biomass reactions (CSM and CSI). The contributions of each class of precursors to these two biomass reactions are shown in Table 1 (see Supplementary Table 2 for a more detailed breakdown). The largest differences in the biomass reactions of both conditions entails the fraction of amino acids, which is approximately doubled in the host as compared to *in vitro* growth on Middlebrook 7H9 medium, which is in accordance with previous predictions (Zimmermann et al., 2017). The fraction of carbohydrates on the other hand, is substantially reduced from 20.1 to 9.9%.

**Table 1:** Composition of the condition-specific biomass reactions.

	**Weight percentage (g/gDw)**	
	**Condition-specific infection, CSI**	Condition-specific medium, CSM^*^
Amino acids	33.2	16.1
Nucleic acids	7.6	8.5
Carbohydrates	9.9	20.1
Lipids	32.5	39.0
Other	16.8	16.2

* *Note that due to rounding of the percentages, the total may not add up to 100%*.

### Simulating Mtb metabolism: balance between growth and enzyme utilization

To predict the *in vivo* metabolic state, reflecting Mtb's intracellular behavior, we compared the performance of five different biomass reactions: the *in vitro* biomass growth reaction (IVB) and a regular biomass growth reaction (REB), both present in sMtb (Rienksma et al., 2014), a biomass reaction representing non-replicating cells (NRC) (Shi et al., 2010), the condition-specific biomass reaction representing growth on Middlebrook 7H9 medium (CSM) and the condition-specific biomass reaction representing growth within the host's phagosome (CSI).

Simulation of the metabolic state of Mtb in the phagosome is complicated by a lack of knowledge on the rate at which nutrients are acquired from the host. However, various studies have shown that the phagosomal environment is likely to be hypoxic (Schnappinger et al., 2003). Therefore, we chose to limit the oxygen uptake rate at a relatively low value of 0.01 mmol gDW^−1^ h^−1^ while keeping unrestricted the uptakes of all other nutrients that were assumed to be present in the host. Even with such a restriction, nutrients were predicted to be taken up in unrealistically large quantities. This behavior can be traced back to anaerobic reactions in the model that result in ATP generation, followed by the artificial generation of oxygen at the cost of high amounts of energy in the form of ATP to ADP conversion. In addition, limiting the oxygen uptake rate all the way to 0 mmol gDW^−1^ h^−1^ resulted in zero flux through the (condition-specific) biomass reaction, and was therefore an unsuitable strategy as well.

To overcome such difficulty, the assumption was made that Mtb utilizes its resources parsimoniously when in a hostile environment. This can be modeled by minimization of enzyme usage while maximizing the flux through the biomass reaction. This bi-objective optimization was performed using a weighted sum method in which the following FBA problem with a weighted objective was solved:

(1.1) $$\begin{array}{r} w=\max\left\{\left(\sum_{i=1}^{n}-f_{e,i}\cdot |v_{e,i}|\right)+f_{b}\cdot v_{b}\right\} \end{array}$$

subject to:

(1.2) S·v = b

(1.3) l ≤ v ≤ u

Wherein *w* is the objective function value, *v*_*e, i*_ represents the flux or rate of reaction *i* catalyzed by at least one enzyme; *f*_*e, i*_ represents the weight factor for reaction *i*; *v*_*b*_ represents the specific growth rate (or biomass reaction flux value), i.e., the flux through one of the five aforementioned (condition-specific) biomass reactions; *f*_*b*_ represents the weight factor for the biomass reaction; *n* is the total number of reactions catalyzed by at least one enzyme; **S** represents the stoichiometric matrix; **v** represents a vector with all fluxes (comprising *v*_*e, i*_ and *v*_*b*_); **b** represents a vector with zeros; **l** represents a vector with lower bounds for all fluxes and **u** represents a vector with upper bounds for all fluxes. The weight factor ratio, *f*_*r*_, between growth and total enzyme utilization is given by:

(2) $$\begin{array}{r} f_{r}=\frac{f_{b}}{\sum_{i=1}^{n}f_{e,i}} \end{array}$$

Each reaction in the model catalyzed by one or multiple enzymes was given the same weight factor (*f*_*e*_) and the weight factor (*f*_*b*_) of the (condition-specific) biomass reaction was varied such that log(*f*_*r*_) varied around a value of 0. A log(*f*_*r*_) value of 0 entails that the numerator and denominator of Equation (2) are of equal size and reflects a balanced weight distribution between minimization of enzyme usage (i.e., maximization of the negative values) and maximization of growth. By changing the weight factor ratio, the relative importance of enzyme usage minimization and biomass reaction maximization changes (Figure 2). If too much weight is put on the minimization of enzyme usage, i.e., *f*_*r*_ becomes too low, the biomass reaction flux value, *v*_*b*_, becomes irrelevant and its value drops to zero, this can be seen at the left hand panel of Figure 2, where the graphs equal zero. The reason that the average flux through enzymatically catalyzed reactions, *v*_*e*_, does not drop to zero when too much emphasis is put on enzyme usage minimization, as can be seen in the middle panel of Figure 2, is because there is a small (0.1 mmol gDW^−1^ h^−1^) growth related maintenance coefficient enforcing a small minimum flux of ATP to ADP conversion.

### Prediction of uptake rates

Figures 3–5 show predicted uptake rates for the five different biomass reactions. As *f*_*r*_ increases, unrealistically high uptake rates are predicted to overcome the restrictions of the oxygen uptake threshold (0.01 mmol gDW^−1^ h^−1^). As can be seen in Figure 2 (black line), the graph representing NRC biomass reaction (non-replicating cells) is slightly shifted as compared to the other objectives. The reason for this is that the total molecular weight of biomass precursors for this objective as obtained from Shi and colleagues is not normalized to one gram. Its value is actually higher, resulting in a larger objective function value at a smaller *f*_*r*_*-*value (Figure 2, right panel). For the four other biomass reactions a balance exists between maximization of growth and minimization of enzyme usage between approximately log(*f*_*r*_) = −0.5 and log(*f*_*r*_) = 0.3 (0.3 ≤ *f*_*r*_ ≤ 2.0). Beyond log(*f*_*r*_) = 0.3 the restrictions of the oxygen uptake threshold are overcome, and *v*_*b*_ and *v*_*i*_-values jump to infinite (for the NRC biomass reaction, this point is reached earlier). An appropriate value for *f*_*r*_ was selected from Figures 2–5 based on the consideration that uptake of asparagine, alanine and glutamate in addition to glycerol-3-phosphate and CO2 from the host is likely to occur during infection (Beste et al., 2013). In addition, nitric oxide is not produced in high amounts by THP-1 cells, and thus not a likely source of nutrition (Fontán et al., 2008), further justifying an *f*_*r*_-value >0.3 [log(*f*_*r*_) > −0.5], when hardly any nitric oxide is predicted to be taken up (Figure 5, right panel). At *f*_*r*_ = 0.8 [log(*f*_*r*_) = −0.1, dashed vertical lines], uptake of glutamate and glycerol-3-phosphate is predicted for all biomass reactions except for NRC, the biomass reaction describing non-replicating cells. For this biomass reaction uptake of glutamate is not predicted. In addition, at this point (*f*_*r*_ = 0.8) uptake of asparagine is predicted for the condition specific biomass reaction of infection (CSI) and predicted to be likely (the average of minimum and maximum uptake rates is above zero) for the other four objectives. The uptake of alanine at this point is predicted to be likely for all five objective functions.

As can be seen in Figures 3–5, the predicted uptake rates are very similar for all five biomass reactions. Therefore, the biomass reaction itself seems of minor importance for the prediction of uptake rates. The uptake of glutamate appears as especially high for a relatively small *f*_*r*_-value, regardless of the chosen biomass reaction.

Beste and colleagues determined that the amino acids asparagine, alanine and glutamate are likely taken up during infection. Acetate- or acetyl-CoA-derived from β-oxidation of host lipids and CO2 is utilized intracellularly and glycerol-3-phosphate could be a potential carbon source as well (Beste et al., 2013). Regardless of the objective used, sMtb is able to reproduce these observations (Figures 3–5). In general, glutamate is taken up at low *f*_*r*_-values, while asparagine becomes more important at higher *f*_*r*_-values. The routes of glutamate toward most metabolic precursors are shorter than those of asparagine, which is predicted to be taken up at a higher *f*_*r*_-value. In this way the change of the uptake rates with the *f*_*r*_-value reflects the metabolic versatility of each component.

Lipid uptake rates show that glycerol-3-phosphate is likely to be taken up, while diacylglycerol and triacylglycerol are possibly taken up. Cholesterol is not predicted to be used as a carbon source at any *f*_*r*_-value, in contrast to mounting evidence that cholesterol plays an important role as a nutrient for Mtb in the host (Wipperman et al., 2014; Rienksma et al., 2015). Currently, the cholesterol degradation pathway of Mtb is partly unknown, therefore only a partial degradation pathway exists in sMtb and the double ringed product (ring C and D of the cholesterol molecule) can only be excreted in sMtb. Partial degradation results in suboptimal yield of energy carrying metabolites derived from the cholesterol molecule compared to other molecules and therefore it is not predicted to be taken up. As knowledge on the cholesterol degradation pathway advances, the complete pathway will eventually be known. Integrating this complete pathway into sMtb will likely yield different results regarding cholesterol uptake.

The prediction of CO_2_ uptake is complicated, as it is a nutrient that is excreted and possibly taken up, unlike the other nutrients in Figures 3–5. With FBA only a prediction of the difference between CO_2_ excretion and uptake can be obtained. On average, CO_2_ is predicted to be excreted throughout the entire *f*_*r*_ range.

### Gene essentiality within the host

Gene essentiality predictions are often used to assess the predictive power of GEMs. Gene essentiality predictions can be simulated with *in silico* gene knock out (KO) mutants and comparing the maximal predicted growth rate of the wild type strain with the KO mutant. A reduction in the predicted specific growth rate of 95% or more is generally accepted as a threshold value for gene essentiality (Beste et al., 2007; Jamshidi and Palsson, 2007; Rienksma et al., 2014).

Here this approach will not provide satisfactory results, as there are too few constraints on the uptake rates of individual nutrients, only on the whole of enzymatically catalyzed reactions, resulting in an excess of unrealistic metabolic routes that could circumvent the deficiency caused by the deletion of the gene. We therefore optimized the aforementioned weighted bi-objective using *f*_*e, i*_ = 0.001 for all *i* with and without deleting the corresponding gene. Afterwards, both results were compared and a reduction of the specific growth rate, *v*_*b*_, by 95% was marked as an essential gene.

These gene essentiality predictions were performed for each of the biomass reactions. We subsequently compared these predictions with experimental data obtained by Mendum et al. (2015) and evaluated the accuracy, sensitivity and specificity of the predictions obtained with each of the five biomass reactions was calculated (Table 2).

**Table 2:** Gene essentiality predictions made using sMtb with five objective functions compared with experimental data obtained 3 and 7 days after infection (Mendum et al., 2015).

	**CSI**		**CSM**		**IVB**		**REB**		**NRC**	
	**3 ds**	**7 ds**	**3 ds**	**7 ds**	**3 ds**	**7 ds**	**3 ds**	**7 ds**	**3 ds**	**7 ds**
TP	47	50	45	47	24	29	45	48	9	10
TN	335	346	343	353	352	365	346	357	419	428
FP	100	97	92	90	83	78	89	86	16	15
FN	222	211	224	214	245	232	224	213	260	251
Accuracy	0.54	0.56	0.55	0.57	0.53	0.56	0.56	0.58	0.61	0.62
Sensitivity	0.17	0.19	0.17	0.18	0.09	0.11	0.17	0.18	0.03	0.04
Specificity	0.77	0.78	0.79	0.80	0.81	0.82	0.80	0.81	0.96	0.97

*TP, true positive; TN, true negative; FP, false positive; FN, false negative; CSI, condition-specific infection reaction; CSM, condition-specific medium reaction; IVB, in vitro biomass reaction; REB, regular biomass reaction; NRC, non-replicating cells reaction*.

## Discussion

We have created condition-specific biomass reactions based on transcript abundance data, thereby ensuring that the obtained biomass compositions represent the organism's needs in the corresponding conditions. By limiting the availability of nutrients to those known or estimated to be present in the phagosome and restricting the uptake of all other nutrients, we were able to capture the metabolic state of Mtb during infection.

Methods such as iMAT (Shlomi et al., 2008), MADE (Jensen and Papin, 2011), or GIMME (Becker and Palsson, 2008), aim at developing condition specific models maximizing the agreement between flux predictions and expression measurements methods. The flexibility of these models is reduced, and this can limit their predictive power. If, for example, certain reactions are perturbed by the effect of drugs, perhaps the system shifts to another metabolic state to accommodate the effect of such perturbation. However, due to the fitting of the gene expression data, it might happen that this effect cannot be accounted for, as the predicted metabolic state is biased to represent the gene expression data. In our approach, we initially constrain the reaction bounds in the model with the gene expression data. The constrained model is used to derive a condition specific biomass reaction. The obtained coefficients of the biomass precursors contain information on the network wide impact of the gene expression data. The constraints in the model are then removed while the newly defined condition specific biomass reaction is used to provide an indirect representation of the metabolic state corresponding to the expression data. Our goal was to retain flexibility in the model, while incorporating the experimental data.

We reasoned that the enzymes encoded by transcripts and involved in metabolism, which were present at a given moment in Mtb, should roughly reflect the flux through these enzymes at that specific condition and time point. Even though transcript abundance is not linearly correlated to enzyme abundance or flux (i.e., the reaction rate of an enzyme) (Bordel et al., 2010), for larger systems, such as pathways or the entire metabolism, a correlation is likely to exist. On average, metabolic transcript abundance data should reflect the optimal quantity of a given enzyme that is sufficient to perform its metabolic task. Production of an excess of metabolic enzymes would be a waste of energy, and thus unfavorable for an organism residing in a hostile environment.

The synthesis routes toward amino acids are predicted to carry more flux during host infection as compared to *in vitro* growth, which is in agreement with other predictions (Zimmermann et al., 2017). This is represented in Table 1 by the higher (doubled) fraction of amino acids required. This suggests that protein synthesis is increased upon infection. Mtb is known to excrete proteins during infection, which could explain this predicted increase (Gengenbacher and Kaufmann, 2012). At the same time, the predicted lipid synthesis requirement is lower during infection than during growth on Middlebrook medium, confirming the lipid-rich diet that Mtb encounters in the host environment (Schnappinger et al., 2003; Gengenbacher and Kaufmann, 2012). Another major difference is the lower carbohydrate synthesis. Following the same reasoning, carbohydrates should be more abundant in the host environment, but it is generally assumed that Mtb has poor access to carbohydrates in this environment (Kalscheuer et al., 2010; Fullam et al., 2016). A possible explanation could be that Mtb does not synthesize carbohydrates as the synthesis of other metabolites are preferred within the host as compared to growth on Middlebrook medium.

We have used a bi-objective optimization approach to simultaneously take into account growth requirements and parsimonious enzyme utilization. The tradeoff between both objectives is apparent in Figure 2. Still the comparison between the uptake profiles in Figures 3–5 led us to conclude that a ratio between both objectives, *f*_*r*_, of 0.8 [corresponding to log(*f*_*r*_) = −0.1] is likely to represent the metabolic state in the host. This suggests that, under these conditions, growth represents a major sink to cellular resources. Here we have selected an equal *f*_*e, i*_ for all enzymatic reactions *i*, however this could be modified to account for differences in enzymes, such as size (molecular weight), activity or degradation rates.

Finally, it should be borne in mind that the transcriptomics data do not represent later infectious states, but a single time point 24 h post infection, before the onset of growth arrest. As can be seen from Figures 3–5, the profiles of uptake rates of different nutrients are quite similar for all five (condition-specific) biomass reactions, even though these reactions are very different. Production of a variety of precursors is apparently possible using a more or less fixed set of nutrients. The predicted combination of nutrients that Mtb acquires during infection is surprising from a modeling point of view. As uptake of one nutrient and subsequent production of energy carrying metabolites (ATP, NADH), biomass precursor(s), and excretion of byproducts, will always be more favorable than that of another metabolite in terms of its potential to sustain growth. The result is that the one nutrient is always favored above another and uptake of multiple nutrients normally does not occur without setting quantitative arbitrary boundaries on uptake rates. This preferential substrate utilization is often regulated at multiple levels, and it should be considered that this type of models does not explicitly account for regulation. Still, the energy and metabolite precursor gain from each nutrient is very balanced using sMtb and the bi-objective optimization, which indicates that enough regulatory information is retained in the transcript data.

A major advantage of the simulations performed within this study is that virtually no assumptions on quantitative uptake rates are required. The only limitation on uptake rates, apart from not allowing uptake of metabolites that are not known or likely to be available in the phagosome, is set on the uptake of oxygen. The phagosome is likely a hypoxic environment (Schnappinger et al., 2003; Gengenbacher and Kaufmann, 2012) and the oxygen uptake rate was therefore (arbitrarily) set to 1% (0.01 mmol gDW^−1^ h^−1^) of the rate used in previous predictions on Mtb metabolism (Jamshidi and Palsson, 2007).

The predictions of essential genes using sMtb and the five different (condition-specific) biomass reactions are not overwhelmingly accurate. In general, the specificity (the correct prediction of non-essential genes) is quite good, but the sensitivity (the correct prediction of essential genes) is very poor. This is rather remarkable, as such a long list of biomass precursors (Supplementary Table 2) is likely to result in a high number of genes predicted to be essential, as there is ample opportunity to disrupt synthesis routes toward many precursors by an *in silico* knockout. Possibly, there are even more metabolic precursors that should be taken into account when creating biomass reactions for Mtb.

Although the biomass reaction representing non-replicating cells, NRC, has the highest accuracy, its sensitivity is the poorest of all biomass reactions, due to its low number of biomass precursors. If one is interested in developing novel therapeutic intervention strategies, the essential genes are arguably the most interesting. In general, the amount of genes that are predicted to be essential is lower than the measured number. This could imply that the list of 108 biomass precursors is still too short. Given that there are 2,500 different lipids identified in Mtb up till now (Sartain et al., 2011), the total number of different metabolites is probably a lot higher. Even if metabolic intermediates are omitted, it is still likely that the total number of biomass precursors is well above 108.

The Mtb genome roughly contains 4,000 genes, of which a quarter has an unknown function (Qin et al., 2013). Model sMtb currently contains 930 genes, which is approximately one-third of the genome having a known function. Extrapolating these figures would mean that there are still an estimated 300 unknown genes in the Mtb genome that are involved in metabolism. So, an estimated quarter of model sMtb is missing. This will undoubtedly affect predictions made with sMtb.

Another, more fundamental problem lies in the possibility that Mtb and the host continuously interact and a steady state is not easily obtained (Garton and Hare, 2013). As the foundation of constraint-based metabolic models is the stoichiometric matrix, wherein a steady state (i.e., synthesis and degradation rates for each metabolite are equal) is assumed for all metabolites, a non-steady state situation might negatively impact the predictions made using sMtb.

The poor prediction of genes essential to survival of Mtb within the host is in stark contrast to *in vitro* predictions previously made using sMtb where accuracies of 80% were reached (Rienksma et al., 2014). Remarkably, the biomass reactions seem to have limited influence on gene essentiality predictions within the host. As the general list of biomass precursors of model sMtb is primarily derived from *in vitro* data of Mtb, or close relatives of Mtb, the list of biomass precursors could be overfitted to *in vitro* growth conditions.

In addition, the condition-specific biomass reactions could be incorrectly inferred. As the biomass precursors are maximized individually one at a time, information regarding their interdependency is not taken into account. One could for example envision maximizing the sum of the flux toward all biomass precursors at the same time, while minimizing the difference between the overall flux profile and the gene expression profile, instead of the approach taken here. Nevertheless, such a strategy is at risk of ignoring precursors and corresponding synthesis pathways that are relatively lowly expressed, and ending up only a few precursors in the biomass reaction.

Another explanation is that important constraints are missing. For example, the influence of metal cofactors such as iron and zinc on the metabolic state is ignored, while these cofactors are crucial for intracellular survival, and many metabolic enzymes do not function without these cofactors (Zondervan et al., 2018).

Taken together, the lack of predictive power of sMtb regarding in-host essential gene predictions could be caused by several problems, one of the most fundamental problems being the absence of a steady state situation. The gene essentiality measurements from Mendum and colleagues show a similar picture, as only 78–80% of the metabolic genes essential for survival are shared between 3 days and 7 days after infection (Mendum et al., 2015). This figure is not strikingly low, but it does point in the direction of a lack of a steady state situation. The effect that a non-steady state situation would have on the predictions of essential genes and the metabolic state is difficult to quantify.

Although Mtb is very similar to *M. bovis* BCG, there are obvious differences. First of all, Mtb is highly pathogenic to humans, while *M. bovis* BCG is a relatively safe organism. From a metabolic point of view, both organisms are highly similar, although there are some notable differences (Lofthouse et al., 2013). Moreover, it is not unimaginable that metabolic differences during infection are highlighted as *M. bovis* BCG is eventually eradicated within human immune cells, while Mtb is able to withstand and thrive within such cells. Another aspect is that the gene essentiality measurements are made 3 days and 7 days after infection while the dual RNA-seq data is derived from an experiment 1 day after infection.

We developed a method of modeling the metabolism of *M. tuberculosis* during infection of the host's immune cells. The method has the advantage that, unlike previously applied host-pathogen modeling approaches (Bordbar et al., 2010), it is virtually free from any artificially placed constraints on metabolite uptake and secretion rates. In addition, our method does not require a pre-composed biomass reaction. The only requirements are: knowledge of nutrient availability, a genome-scale dataset of transcript abundances (such as an RNA-sequencing dataset), a detailed list of biomass precursors, and a genome-scale constraint-based model of metabolism. A relatively small amount of data is required for this method, and it is therefore suited to explore metabolic states of microorganisms in difficult to access environments where an efficient usage of resources is likely to occur.

Our method allows accurate prediction of nutrients from the host, apart from cholesterol uptake, which was not predicted to take place, likely due to lack of knowledge on the complete degradation pathway. A doubled amino acid synthesis requirement was predicted using our method, suggesting an increased synthesis rate of proteins relative to other metabolic precursors during host infection. Lipid synthesis was predicted to decrease during infection, confirming the predominant lipid diet encountered by Mtb within the host.

Flux predictions obtained with the condition specific biomass reactions, without any further constraints show poor correlation with the transcriptomics data (lower than 0.1). This value is similar to the values obtained using the other four biomass reactions. Poor correlation between transcriptomics data and proteomics measurements has been shown in a wide number of publications (Maier et al., 2009; Payne, 2015; Edfors et al., 2016). In addition, accurate predictions would also require inclusion of enzyme turnover data (Sánchez Benjamín et al., 2017). This further confirms that fitting the model to the gene expression data might lead to an over-constrained model.

It is important to notice that during the onset of infection not only the bacterium undergoes metabolic changes, but also the host environment it thrives in most likely undergoes changes as the host responds to infection. This interplay between the host and the pathogen has not been taken into account as here only the bacterium is modeled. Another reason for the inaccurate gene essentiality predictions could be that many enzymes play additional roles in the synthesis of precursors that are not required during *in vitro* growth or that the list of precursors is not comprehensive. The latter explanation would be plausible, as the predictions on nutrient uptake are quite accurate, suggesting that nutrient uptake is driven by energy efficiency constraints.

## Author contributions

RR drafted the manuscript, conceived the study and performed the data analysis. MS-D participated in drafting the manuscript and helped with the data analysis. PS and VM participated in drafting the manuscript. All authors contributed to the study design and critically read, revised and approved the manuscript.

### Conflict of interest statement

The authors declare that the research was conducted in the absence of any commercial or financial relationships that could be construed as a potential conflict of interest.
