# Supplementary material for: Modeling the Metabolic State of Mycobacterium tuberculosis Upon Infection
Source: Front Cell Infect Microbiol. 2018 Aug 3;8:264. doi: 10.3389/fcimb.2018.00264 (PMC6085482; doi:10.3389/fcimb.2018.00264)
Supplement: Supplementary file 1 [file Table_1.DOCX]

Nutrient availability

Listed below are the available nutrients for generation of condition-specific biomass reactions. Presence of a nutrient is indicated by an 'x', M denotes medium (the nutrients available for Mtb in Middlebrook 7H9 medium), I denotes infection (the nutrients available for Mtb in the phagosome) (Schnappinger, Ehrt et al. 2003, Beste, Nöh et al. 2013, Wipperman, Sampson et al. 2014).

|  | nutrient | nutrient name in sMtb | M | I |
| --- | --- | --- | --- | --- |
| 1 | 9-octadecenoate | 9OCTADECENOATE | x |  |
| 2 | alanine | ALA |  | x |
| 3 | aspartate | ASP |  | x |
| 4 | asparagine | ASN |  | x |
| 5 | biotin | BIOTIN | x |  |
| 6 | calcium ion | CA | x | x |
| 7 | carbon dioxide | CO2 | x | x |
| 8 | citrate | CIT | x |  |
| 9 | chloride ion | CL | x | x |
| 10 | cholesterol | CHOLESTEROL |  | x |
| 11 | cobalt ion | COII | x | x |
| 12 | copper ion | CU | x | x |
| 13 | diacylglycerol | DAG |  | x |
| 14 | iron 2+ ion | FE2 | x | x |
| 15 | iron 3+ ion | FE3 | x | x |
| 16 | glycerol | GL | x |  |
| 17 | glycerol-3-phosphate | GL3P |  | x |
| 18 | glucose | GLC | x |  |
| 19 | glutamate | GLU | x | x |
| 20 | water | H2O | x | x |
| 21 | proton | H | x | x |
| 22 | potassium | K | x | x |
| 23 | magnesium | MG | x | x |
| 24 | manganese | MN | x | x |
| 25 | molybdate | MOLYBDATE |  | x |
| 26 | sodium | NA | x | x |
| 27 | ammonia | NH3 | x |  |
| 28 | nitric oxide | NO |  | x |
| 29 | oxygen | O2 | x | x |
| 30 | phosphate | PI | x | x |
| 31 | pyridoxine | PYRI | x |  |
| 32 | sulfate | SLF | x | x |
| 33 | triacylglycerol | TAG |  | x |
| 34 | zinc | ZN | x | x |

**References**

Beste, Dany J. V., K. Nöh, S. Niedenführ, Tom A. Mendum, Nathaniel D. Hawkins, Jane L. Ward, Michael H. Beale, W. Wiechert and J. McFadden (2013). "13C-Flux Spectral Analysis of Host-Pathogen Metabolism Reveals a Mixed Diet for Intracellular *Mycobacterium tuberculosis*." Chemistry & Biology **20**(8): 1012-1021.

Schnappinger, D., S. Ehrt, M. I. Voskuil, Y. Liu, J. A. Mangan, I. M. Monahan, G. Dolganov, B. Efron, P. D. Butcher, C. Nathan and G. K. Schoolnik (2003). "Transcriptional Adaptation of *Mycobacterium tuberculosis* within Macrophages: Insights into the Phagosomal Environment." J Exp Med **198**(5): 693-704.

Wipperman, M. F., N. S. Sampson and S. T. Thomas (2014). "Pathogen roid rage: Cholesterol utilization by *Mycobacterium tuberculosis*." Critical Reviews in Biochemistry and Molecular Biology: 1-25.
