# Supplementary material for: Modeling the Metabolic State of Mycobacterium tuberculosis Upon Infection
Source: Front Cell Infect Microbiol. 2018 Aug 3;8:264. doi: 10.3389/fcimb.2018.00264 (PMC6085482; doi:10.3389/fcimb.2018.00264)
Supplement: Supplementary file 2 [file Table_2.DOCX]

Biomass precursors

A list of possible biomass precursors obtained from model sMtb. The coefficients of the five different objective functions are given in the columns CSI, CSM, IVB, REB, and NRC. Zeros are omitted.

|  |  | coefficient value ( · 10^3^) | | | | |
| --- | --- | --- | --- | --- | --- | --- |
| Precursor name | Precursor name in sMtb | CSI | CSM | IVB | REB | NRC |
| 5MTR | 5-Methylthio-D-ribose | 19 | 16 |  |  |  |
| AC1PIM1 | acyl phosphatidylinositol mannoside mannose | 16 | 26 | 3 | 2 |  |
| AC1PIM2 | acyl phosphatidylinositol mannoside di-mannose | 12 | 22 | 2 | 1 |  |
| AC1PIM6 | acyl phosphatidylinositol mannoside hexa-mannose | 6 | 13 | 3 | 2 |  |
| AC2PIM2 | di-acyl phosphatidylinositol mannoside di-mannose | 11 | 20 | 3 | 2 |  |
| AC2PIM6 | di-acyl phosphatidylinositol mannoside hexa-mannose | 6 | 12 | 3 | 2 |  |
| AGA6P | N-Acetyl-D-glycosamine 6-phosphate |  | 15 |  |  |  |
| ALA | L-Alanine | 379 | 95 | 218 | 218 |  |
| AMYL | Amylose monomer | 40 | 68 | 372 | 235 |  |
| ARABINOGALACTANPEPTIDOGLYCAN | arabinogalactan-peptidoglycan large cell wall complex | <1 | <1 | 2 | 2 |  |
| ARAFDPP | arabinofuranose-decaprenylphosphate | 4 | 5 | 53 | 39 |  |
| ARG | L-Arginine | 48 | 26 | 115 | 115 |  |
| ASN | L-Asparagine | 315 | 26 | 35 | 35 |  |
| ASP | L-Aspartate | 364 | 26 | 89 | 89 |  |
| BIOTIN | Biotin | 8 | 29 |  |  |  |
| C78MYCOLATEPP | C78-alpha-mycolate-PP | 3 | 4 | 40 | 30 |  |
| CAL | Cardiolipin | 20 | 12 | 12 | 9 |  |
| COA | Coenzyme A | 9 | 9 | 16 | 13 |  |
| COBCO | Cobamide coenzyme |  |  |  |  |  |
| COBIII | Aquacob(III)alamin |  |  |  |  |  |
| CTP | CTP | 20 | 24 | 17 | 17 |  |
| CYS | L-Cysteine | 30 | 28 | 16 | 16 |  |
| DATP | dATP | 15 | 13 | 10 | 10 |  |
| DCTP | dCTP | 20 | 24 | 19 | 19 |  |
| DGTP | dGTP | 14 | 17 | 19 | 19 |  |
| DHA | Glycerone | 92 | 145 |  |  |  |
| DHXP | 7,8-dihydroxanthopterin | 17 | 20 |  |  |  |
| DIMAM | phthiodiocerol dimycocerosate DIMA membrane associated | 16 | 14 |  | 1 |  |
| DIMBM | phthiodiolone dimycocerosate DIMB membrane associated | 19 | 14 |  | 1 |  |
| DLIPO | Dihydrolipoamide | 7 | 6 | 1 | 1 |  |
| DTTP | dTTP | 17 | 22 | 10 | 10 |  |
| ETHA | Ethanolamine | 30 | 28 |  |  |  |
| F420 | Coenzyme F420 | 6 | 9 |  |  |  |
| FAD | FAD | 6 | 9 | 16 | 13 |  |
| FE2 |  |  |  | 1 | 1 |  |
| FE3 |  |  |  | 1 | 1 |  |
| FERI |  |  |  | 1 | 1 |  |
| FERO |  |  |  | 1 | 1 |  |
| FMN | FMN | 10 | 13 |  | 22 |  |
| G3PE | sn-glycero-3-Phospoethanolamine | 30 | 28 |  |  |  |
| GDPFUC | GDP-L-fucose |  |  | 38 | 24 |  |
| GDPMAN | GDP-mannose | 11 | 17 | <1 | <1 |  |
| GL | Glycerol | 93 | 29 | 25 | 18 |  |
| GLC | D-Glucose | 53 | 161 |  | 466 |  |
| GLN | L-Glutamine | 224 | 163 | 138 | 138 | 41 |
| GLU | L-Glutamate | 316 | 381 |  | 162 | 41 |
| GLUCAN | D-glucan monomer | 53 | 161 | 372 | 235 |  |
| GLY | Glycine | 30 | 28 | 158 | 158 |  |
| GSSG | Glutathione disulfide | 8 | 7 |  |  |  |
| GTP | GTP | 14 | 17 | 17 | 17 |  |
| HBUTCOA | (S)-3-Hydroxybutanoyl-CoA | 8 | 9 |  |  |  |
| HEMEFE2 | Heme O | 11 | 15 | 1 | 1 |  |
| HEMEFE3 | Heme A | 11 | 15 | 1 | 1 |  |
| HIS | L-Histidine | 26 |  | 34 | 34 |  |
| HYDROXYPHTHIOCERANOYLCOA | hydroxyphthioceranoyl-CoA | 7 | 9 |  | 6 |  |
| ILE | L-Isoleucine | 96 | 26 | 64 | 64 |  |
| LEU | L-Leucine | 131 | 53 | 148 | 148 |  |
| LYS | L-Lysine | 104 |  | 32 | 32 |  |
| MAN | D-Mannose | 53 | 161 | 277 | 175 |  |
| MBTC18ANOYL | Mycobactin with octadecanoyl group | 14 |  |  | <1 |  |
| MBTC18ENOYL | Mycobactin with octadecenoyl group | 14 |  |  | <1 |  |
| MBTMAL | Mycobactin with malonyl group | 15 |  |  | <1 |  |
| MET | L-Methionine | 53 | 26 | 29 | 29 |  |
| MGD | Molybdopterin guanine dinucleotide |  |  |  |  |  |
| MQ | Menaquinone | 5 |  |  |  |  |
| MOLYBDENUM | Molybdenum cofactor |  |  |  |  |  |
| MOLYBDOPTERIN | Molybdopterin |  |  | 1 | 1 |  |
| MQ |  |  |  | 14 | 12 |  |
| MTR1P | S-Methyl-5-thio-D-ribose 1-phosphate |  | 10 | 1 | 4 |  |
| MYCOLIPANOATE | Mycolipanoate |  |  |  | <1 |  |
| MYCOLIPDIENOATE | Mycolipdienoate |  |  |  | <1 |  |
| MYCOLIPENOATE | Mycolipenoate |  |  |  | <1 |  |
| MYCOTHIOL | Mycothiol | 13 | 25 | 18 | 13 |  |
| NACYS | N-acetyl-L-cysteine | 30 | 28 |  |  |  |
| NAD | NAD+ | 9 | 9 | 18 | 15 |  |
| NADP | NADP+ | 9 | 9 | 16 | 14 |  |
| PE | Phosphatidylethanolamine | 30 | 28 | 4.9 | 3.6 |  |
| PENTAMETHYLTRICONTANOYLCOA | PENTA-METHYL-TRICONTANOYL-COA | 7 | 9 | 0.1 | 0.1 |  |
| PHBADI | p-hydroxybenzoic acid derivative I | 14 | 24 |  |  |  |
| PHBADII | p-hydroxybenzoic acid derivative II |  |  |  |  |  |
| PHDIMAM | Phenol-phthiodiocerol dimycocerosate PHDIMA membrane associated |  |  |  | 3 |  |
| PHDIMBM | Phenol-phthiodiolone dimycocerosate PHDIMB membrane associated |  |  |  | 3 |  |
| PHE | L-Phenylalanine | 30 | 24 | 43 | 43 |  |
| PPGPPP | Guanosine 3-diphosphate 5-triphosphate | 12 | 14 | 1 | 1 |  |
| PPM | D-Mannosyl-1-phosphoundecaprenol | 4 | 4 |  | 26 |  |
| PRO | L-Proline | 69 | 77 | 92 | 92 |  |
| PURI5P | Pseudouridine 5-phosphate | 29 | 26 |  |  |  |
| PYRI | Pyridoxine | 38 | 29 |  |  |  |
| PYRM5P | Pyridoxamine phosphate | 31 | 23 |  |  |  |
| RIB | Ribose | 55 | 95 | 81 | 51 |  |
| SAM | S-Adenosyl-L-methionine | 13 | 9 | 42 | 35 |  |
| SDLIPO | S-Succinyldihydrolipoamide | 7 | 6 |  |  |  |
| SER | L-Serine | 30 | 28 | 89 | 89 |  |
| SIROHEME | Siroheme | 19 | 28 | 1 | 1 |  |
| SL1 | sulfolipid-1 | 7 | 8 |  | 6 |  |
| SPRMD | Spermidine | 19 | 26 | 1 | 4 |  |
| SRH | S-Ribosyl-L-homocysteine | 23 | 26 |  | 71 |  |
| TAG | Triacylglycerol | 37 | 35 | 97 | 71 | 16 |
| THF | Tetrahydrofolate | 10 | 15 | 1 | 1 |  |
| THFG | Tetrahydrofolyl-[Glu](2) | 10 | 14 |  |  |  |
| THIPP | Thiamin diphosphate |  | 12 | 1 | 1 |  |
| THR | L-Threonine | 129 | 26 | 92 | 92 |  |
| TRE6P | alpha,alpha-Trehalose 6-phosphate | 20 | 45 |  | 18 |  |
| TREHALOSEDIMYCOLATE | alpha,alpha-Trehalose 6,6-bismycolate | 5 | 5 |  |  | 12 |
| TREHALOSEMONOMYCOLATE | alpha,alpha-Trehalose 6-mycolate | 9 | 9 |  | 30 |  |
| TRP | L-Tryptophan | 19 | 24 | 22 | 22 |  |
| TYR | L-Tyrosine | 30 | 24 | 31 | 31 |  |
| UDPGLCUR | UDP-glucuronate | 15 | 24 |  |  |  |
| UDPNAM | UDP-N-acetylmuramate | 13 | 22 | 63 | 40 |  |
| UTP | UTP | 21 | 26 | 9 | 9 |  |
| VAL | L-Valine | 150 | 90 | 137 | 137 |  |
| XYLU | D-Xylulose | 33 | 24 |  |  |  |
