# Supplementary material for: Modeling the Metabolic State of Mycobacterium tuberculosis Upon Infection
Source: Front Cell Infect Microbiol. 2018 Aug 3;8:264. doi: 10.3389/fcimb.2018.00264 (PMC6085482; doi:10.3389/fcimb.2018.00264)
Supplement: Supplementary file 5 [file Data_Sheet_3.DOCX]

Summary of changes to model sMtb

Model sMtb was obtained from (Rienksma, Suarez-Diez et al. 2014) and the following was changed:

1. The gene association of reaction CYA is changed to: Rv1625c OR Rv2435c OR Rv1264 OR Rv1318c OR Rv1319c OR Rv1320c.
2. Reaction MQO was added, with gene association Rv2852c (gene mqo, malate:menaquinone oxidoreductase).
3. Metabolite abbreviation MK was changed to MQ (menaquinone) and metabolite abbreviation DMK was changed to DMQ (2-demethyl menaquinone).
4. Menaquinol (MQH2) was added as a metabolite.
5. Reaction COCO2 was replaced by reaction COX, which is formulated as: 1 CO[c] + 1 H2O[c] + 1 MQ[c] --> 1 CO2[c] + 1 MQH2[c], with gene association: Rv0368c AND Rv0369c AND Rv0370c AND Rv0371c AND Rv0372c AND Rv0373c AND Rv0374c AND Rv0375c AND Rv0376c.
6. Reaction SDHA was split into three reactions: SDH1, SDH2, and FRD. SDH2 is the same as the previous reaction SDHA, apart from the gene association, which was changed to: Rv3318 AND Rv3319 AND Rv3316 AND Rv3317.
7. Reaction SDH1 is formulated as: 1 MQ[c] + 1 SUCC[c] --> 1 MQH2[c] + 1 FUM[c], with gene association: Rv0247c AND Rv0248c AND Rv0249c.
8. Reaction FRD is formulated as: 1 MQH2[c] + 1 FUM[c] --> 1 MQ[c] + 1 SUCC[c], with gene association: Rv1552 AND Rv1553 AND Rv1554 AND Rv1555.
9. Reaction CYDA was replaced by reaction QCRCTA, wich is formulated as: 2 MQH2[c] + 1 O2[c] + 6 H[c] --> 2 MQ[c] + 2 H2O[c] + 6 Hr[e], with gene association: Rv2194 AND Rv2195 AND Rv2196 AND Rv1451 AND Rv1456c AND Rv3029c AND Rv3028c AND Rv2200c AND Rv3043c AND Rv2193.
10. Reaction CYDB was replaced by reaction CYD, wich is formulated as: 2 MQH2[c] + 1 O2[c] --> 2 MQ[c] + 2 H2O[c], with gene association: Rv1623c AND Rv1622c AND Rv1621c AND Rv1620c.
11. Metabolite Q en QH2 were changed to MQ and MQH2 respectively, for the following reactions: NDH, NARG, NUO and GLPD2. The metabolites ubiquinone (Q) and ubiquinol (QH2) were subsequently removed from the model.
12. Reaction HYD was added, which is formulated as: 1 H2[c] + 2 NAD[c] --> 2 NADH[c], with gene association: Rv0081 AND Rv0082 AND Rv0083 AND Rv0084 AND Rv0085 AND Rv0086 AND Rv0087 AND Rv0088.
13. Metabolite H2 (hydrogen gas) was added to the model, together with a transport reaction (TH2), which is formulated as: 1 H2[e] --> 1 H2[c].
14. Reaction HEOX was added, which is formulated as: 1 HEMEFE2[c] + 1 O2[c] --> 1 HEMEFE3[c] + 1 H2O[c].
15. Reaction TDAG was added, which is formulated as: 1 DAG[e] --> 1 DAG[c].
16. Reaction TPA was added, degrading triphosphate to diphosphate, which is formulated as: 1 PPPI[c] + 1 H2O[c] --> 1 PPI[c] + 1 PI[c].
17. The reversibility of reaction PCKA was changed from irreversible to reversible.

**References**

Rienksma, R. A., M. Suarez-Diez, L. Spina, P. J. Schaap and V. A. P. Martins dos Santos (2014). "Systems-level modeling of mycobacterial metabolism for the identification of new (multi-)drug targets." Seminars in Immunology **26**(6): 610-622.
